# Assessing Heterogeneity in Students’ Visual Judgment: Model-Based Partitioning of Image Rankings

**DOI:** 10.3389/fpsyg.2022.881558

**Published:** 2022-08-10

**Authors:** Miles Tallon, Mark W. Greenlee, Ernst Wagner, Katrin Rakoczy, Wolfgang Wiedermann, Ulrich Frick

**Affiliations:** ^1^Department of Experimental Psychology, University of Regensburg, Regensburg, Germany; ^2^HSD Research Centre Cologne, HSD University of Applied Sciences, Cologne, Germany; ^3^Academy of Fine Art Munich, Munich, Germany; ^4^Institute for School Education and Empirical Educational Research, Justus-Liebig University, Gießen, Germany; ^5^Missouri Prevention Science Institute and Department of Educational, School, and Counseling Psychology, University of Missouri, Columbia, KY, United States

**Keywords:** visual abstraction, assessment, Bradley–Terry model, model-based partitioning, ranking, art education, visual literacy

## Abstract

Differences in the ability of students to judge images can be assessed by analyzing the individual preference order (ranking) of images. To gain insights into potential heterogeneity in judgement of visual abstraction among students, we combine Bradley–Terry preference modeling and model-based recursive partitioning. In an experiment a sample of 1,020 high-school students ranked five sets of images, three of which with respect to their level of visual abstraction. Additionally, 24 art experts and 25 novices were given the same task, while their eye movements were recorded. Results show that time spent on the task, the students’ age, and self-reported interest in visual puzzles had significant influence on rankings. Fixation time of experts and novices revealed that both groups paid more attention to ambiguous images. The presented approach makes the underlying latent scale of visual judgments quantifiable.

## Introduction

This study is part of a larger research project on the assessment of Visual Literacy (VL) and how VL can be fostered in art education (Frick et al., 2020). VL, a core competency in art education, comprises the ability to evaluate artwork with respect to aesthetic value. The Common European Framework of Reference for Visual Literacy (CEFR-VL; Wagner and Schönau, 2016) defines *judging* (or evaluating) images as the ability to formulate a justified statement or estimation about images and artistic creations. We define visual abstraction as a prerequisite for aesthetic judgment and as a latent variable in a visual judgement task. The method described here contributes to determine essential variables that impact the judgment of latent image features (exemplified by visual abstraction) and in return might help teachers detect and promote students’ development of artistic skills. Furthermore, identifying critical variables that influence students’ visual judgments may be important for empirical art education research. The aim of the present study is to investigate students’ ability, on the one hand, as well as that of experts and novices, on the other hand, to judge images based on the level of perceived visual abstraction, while placing a focus on the identification of biographical and psychological characteristics that influence these judgments. Aesthetic judgments are not only influenced by the properties of the items being judged but they are influenced by additional factors such as expertise and personal experience (Child, 1965; Nodine et al., 1993; Jacobsen, 2004; Hayn-Leichsenring et al., 2020; McCormack et al., 2021). For example, Chamorro-Premuzic and Furnham (2004) showed that university students with higher interests in art tend to score higher on art judgment tasks and that these judgments were significantly related to both personality and intelligence.

Every artwork, whether figurative or not, is a form of abstraction (Witkin, 1983; Gortais, 2003). However, the measurement of the perceived level of visual abstraction in artworks remains challenging. A study that specifically tried to measure the perceived level of visual abstraction used visual analog scales to rate artworks as “abstract” and found contrast effects due to sequential presentation of high vs. low abstract paintings on participants’ judgments (Specht, 2007). Other studies explored the preference judgment of abstract art measured by Likert-scale ratings and revealed a preference for the artists’ original compositions (McManus et al., 1993; Furnham and Rao, 2002). Efforts to quantify visual abstraction in artworks were also made by Chatterjee et al. (2010): their Assessment of Art Attributes instrument (AAA) includes “abstraction” as a conceptual-representational attribute. The level of abstraction is measured *via* a Likert-scale rating and training slides with example images as anchors. Another assessment tool, the Rating Instrument for Two-Dimensional Pictorial Works (RizbA; Schoch and Ostermann, 2020), consists of 26 six-point Likert-scale items, including two questions regarding the mode of concrete and abstract representation.

However, when underlying image features are latent (e.g., the extent to which a given image is abstract) metric scales may fall short when asked to judge these items by, for example, assigning a number from 1 to 10. Typical disadvantages of the use of such absolute measures may include anchor effects (Furnham and Boo, 2011) and end-aversion bias (Streiner and Norman, 2008) among others (Choi and Pak, 2005). It is often easier to compare items to each other, e.g., in a series of paired comparison (PC) tasks. Such comparative measures can be analyzed with Bradley–Terry (BT) models (Bradley and Terry, 1952), also referred to as Bradley–Terry-Luce models. BT models are a popular method to uncover a latent preference scale of objects/items from paired comparison data (Cattelan, 2012). For example, BT models are frequently used to determine the best sport teams (Cattelan et al., 2013), to analyze consumer-specific preferences (Dittrich et al., 2000), or to determine the perceived harm of psychotropic substances (Wiedermann et al., 2014). When multiple objects (images) are compared simultaneously, ranking tasks (e.g., ranking images according to their level of abstraction) constitute valuable alternatives to PCs. Ranking data can then be transformed into derived PC patterns (Francis et al., 2010).

The present study focuses on potential heterogeneity in visual judgments. Potential differences in visual judgments were evaluated in two samples: a sample of high-school students and an additional sample comprising art experts (art educators, artists, designers) and novices (art laypersons). In the student sample, self-reported visual skills and demographic variables are used to detect potential differences in students’ performance to rank different sets of images based on their level of visual abstraction. In the experts and novices sample eye movements were additionally recorded during the image ranking task. Eye movement indicators are used to analyze the distribution of attention (Jarodzka et al., 2017; Brams et al., 2019). Eye tracking, in particular as an exploratory tool, can enhance the multidisciplinary field of VL research, as it visualizes cognitive processes involved in visual problem solving and art perception (Brumberger, 2021). Visualizing the solution process with VL-expert’s and novices’ eye-movements can be used to uncover cognitive processes that differ between the expert and novice groups and may further reveal difficult or ambiguous image sets.

This study uses model-based partitioning as a method to analyze what underlies the variability in visual judgments. We use a recently published approach that combines Bradley–Terry (BT) models with model-based recursive partitioning (trees) to detect preference heterogeneity in subgroups (Wiedermann et al., 2021). BT models are well-suited for (art) educational assessment tasks, in which students are instructed to rank images based on given criteria. From a methodological perspective the use of BT models in combination with recursive partitioning is studied for its potential when applied to art education assessment. The reason for this is that conventional statistical analysis of interaction effects may fall short when tasked to address the complex moderation processes of visual judgments. The method used here enables researchers to differentiate between the effects of student characteristics and learning interventions on latent preference rankings more closely. The study addresses the following research questions: What effects do self-reported visual skills and student characteristics have on the order of images when they are ranked according to visual abstraction? Do VL-experts and novices differ in their ranking patterns and solution strategies?

## Materials and Methods

### Subjects and Stimuli

Sample I comprised 1,020 students of which 987 worked on the ranking tasks and filled out the questionnaire. A total of 52 classes (9th to 13th grade) from 29 schools in Germany took part in the study. Two classes did not receive the questionnaire and one class could not be offered the ranking task due to technical difficulties. To control for potentially nested effects of classrooms, intraclass correlation coefficients (ICCs) for intended rankings were calculated on each image set. Due to low values (ICCs range from 0.01 to 0.03, for calculations see Chakraborty and Sen, 2016), no multi-level adjustments were necessary. Overall, 52% of participants were female, the average age was 15.34 years (*SD* = 2.96). Schools were recruited in the federal states of Hessen, North-Rhine Westphalia, Schleswig-Holstein, and Rhineland Palatinate *via* leaflets, letters and recommendations. Data collection was conducted in classrooms with up to 30 students (*M* = 20.8, *SD* = 5.10). The image ranking task was part of a VL assessment test battery, including demographic questions, art grade, and the following questions regarding artistic ability and self-perceived art skills (S1–S5):

- If you had to rank all of your classmates according to their abilities in the subject of art, where would you rank yourself? [S1; scored 1 (as one of the worst) to 5 (as one of the best)]
- How good are you at art in general? [S2; scored 1 (very bad) to 5 (very good)]
- How good are you in theoretical content (art theory; e.g. interpreting pictures, understanding art history)? [S3; scored 1 (very bad) to 5 (very good)]
- How good are you in practical activities in art class (e.g., painting, drawing, drafting, and designing)? [S4; scored 1 (very bad) to 5 (very good)]
- Compared to your skills in other school subjects: How well do you rate your art skills? [S5; scored 1 (much worse) to 5 (much better)]

Additionally the following self-reported visual skills were rated on a scale from 1 (strongly disagree) to 4 (strongly agree): Photographic memory (PM): “I have a ‘photographic memory’”; Spatial orientation (SO): “When I see a photograph of a geometric object, I can imagine what it looks like from behind”; Long-term memory (LM): “I can remember small details in pictures”; Imagination (IM): “I can easily picture things mentally”; and Interest in visual puzzles (IP): “I like to solve picture puzzles.”

Sample II comprised 51 participants of which 49 participants had qualitatively sufficient eye-tracking data to be included for further analyses. Experts and novices were screened based on their experience and interest or profession in the visual arts. The expert group (*n* = 24) consisted of photographers, artists, designers, and art students. The novice group (*n* = 25) consisted of students and adults from various educational institutions who were not associated with academic or professional work in the visual arts. The mean age of participants were *M* = 29.08 years (*SD* = 12.55). The participants in sample II were assessed individually in seminar or laboratory rooms (e.g., at the Academy of Fine Arts in Munich).

In sample I school classes were offered a lump sum of 100€ as collective compensation. In sample II student participants each received 20€ as compensation. Participants from the expert group, who were generally interested in the subject of visual literacy and eye tracking, took part without further incentive. All participants and their legal representatives, respectively, gave written consent before participating in this study. The study was approved by the Ethics Committee of Research of the Leibniz Institute for Research and Information in Education, Frankfurt am Main (DIPF, 01JK1606A).

### Ranking Task

We used images with varying level of visual abstraction, i.e., image sets that represent the gradual process of transforming figurative artwork to non-figurative artwork (Viola et al., 2020). As every work of art uses some level of abstraction, many artworks could be investigated. Therefore images were curated (or created) by visual arts professionals from the board of the European Network for Visual Literacy (ENViL). Image sets were chosen based on the likelihood of being discussed in art class, representing a varying degree of abstraction.

Overall, five ranking tasks were presented on Android tablets with 10.1 inch screen size (Andrews et al., 2018). Subjects ranked five images, resulting in a total of $\left(\begin{array}{c} 5\\ 2 \end{array}\right)=10\;$ paired comparisons for each set of images (with a total of 5! = 120 possible combinations; see Table 1). All participants were presented with the same initial ordering of images and were instructed to rank each image according to two characteristics presented below each image set. The image sets included:

1. geometric figures
2. dogs
3. bull images, inspired by Pablo Picasso’s *Bull* lithographs (MacTaggart, 2021)
4. Mondrian trees
5. salt packages (only presented in sample I)

**Table 1:** Design structure of the loglinear BT pattern model for rankings obtained from *J* = 5 images.

Rankings	Paired comparison (PC) patterns										Counts	Model parameters					
	*y*_12_	*y*_13_	*y*_14_	*y*_15_	*y*_23_	*y*_24_	*y*_25_	*y*_34_	*y*_35_	*y*_45_		Intercept	*x*_1_	*x*_2_	*x*_3_	*x*_4_	*x*_5_
a b c d e	1	1	1	1	1	1	1	1	1	1	*n*_1_	1	4	2	0	−2	−4
b a c d e	−1	1	1	1	1	1	1	1	1	1	*n*_2_	1	2	4	0	−2	−4
c a b d e	−1	−1	1	1	–1	1	1	1	1	1	*n*_3_	1	2	0	4	−2	−4
…	…	…	…	…	…	…	…	…	…	…	…	…	…	…	…	…	…
c e d b a	−1	−1	−1	−1	−1	−1	−1	1	1	−1	*n*_118_	1	−4	−2	4	0	2
d e c b a	−1	−1	−1	−1	−1	−1	−1	−1	−1	1	*n*_119_	1	−4	−2	0	4	2
e d c b a	−1	−1	−1	−1	−1	−1	−1	−1	−1	−1	*n*_120_	1	−4	−2	0	2	4

Rankings are transformed into paired comparison (PC) patterns; the *y*’s represent obtained PCs (*y_jk_* = 1 if *j* > *k* and *y_jk_* = −1 if *k* > *j*), each possible combination of *J*! is then counted as observed frequencies in column “counts,” and *x*’s are auxiliary variables used to estimate model parameters indicating how often *j* was preferred minus how often *j* was not preferred.

**Figure 1:**
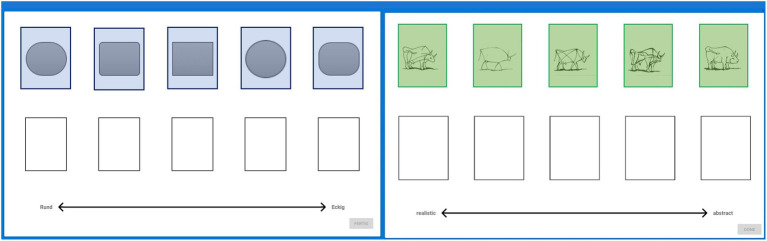
Ranking items “geometric figures” and “bull images.” Each image (left: geometric figures, right: bull images) needs to be placed into an empty slot below to form a ranking (left: from round to edgy and right: from realistic to abstract). Areas of Interest (AOIs) in blue and green were not visible by subjects.

Images had to be ranked according to the following image characteristics: starting with an introductory item to make sure that participants understood the task (“geometric figures”), from round to edgy, the items “dogs,” “bull images,” and “Mondrian trees” had to be ranked by level of visual abstraction; from most realistic to most abstract. Additionally, as a control condition, perceived expensiveness (from cheap to expensive) of items (“salt packages”) was assessed. In contrast to the evaluation of image abstraction, rankings based on unknown prices should stand out as visible outliers compared to the other rankings. This was used in an attempt to investigate potential uncertainty of judgments and how this variability may affect the BT ranking results on group level. The ordering (a > b > c > d > e) of images was consensually decided by VL experts from ENViL. Participants used a touchscreen to select and drop each image into empty slots presented below the images (see Figure 1). The image rankings are then analyzed to gain insights into the possible effects of the participant characteristics on the perceived judgment of abstraction.

### Eye Tracking

Each participant in sample II wore eye-tracking glasses (SMI ETG 2w Analysis Pro) during task performance. Eye movements were recorded at 60 Hz. A 3-point calibration was performed on the tablet for each participant. All participants had normal or corrected to normal eyesight. Fixations were mapped onto corresponding reference images using SMI fixation-by-fixation semantic gaze mapping (Vansteenkiste et al., 2015). Areas of Interest (AOIs) were drawn on each image to assess fixation time and number of fixations spend on each image. Eye-movement events were determined by the SMI velocity-based algorithm (Engbert et al., 2016). Eye-tracking data, i.e., number of fixations, fixation duration and heatmaps were analyzed with SMI BeGaze version 3.7. Heatmaps are used as exploratory tools to investigate eye movements (Bojko, 2009) supplementing the BT models.

### Data Analytic Strategy

We used Bradley–Terry (BT) models as the basis for recursive partitioning. The BT model is a probability model that can be used to predict the outcome of paired comparisons and to obtain (cardinal) preferences values for all items (images) on a latent scale (Bradley and Terry, 1952). Here, “preference” refers to the judgment of image characteristics (e.g., abstractness) by each participant. Under this model one considers a set of J objects which are presented in pairs. The probability of preferring item *j* over item *k* can be described as


(1) $$p_{j>k}=\frac{\pi_{j}}{\pi_{j}+\pi_{k}},$$

with $\pi_{j}\geq \mathrm{and}\overset{J}{\underset{j=1}{\sum }}\pi_{j}$ representing “worth” of the item j, quantifying the position of the item j on a standardized latent scale from 0 to 1. BT models can be fitted as loglinear Bradley–Terry models (LLBT; Sinclair, 1982; Dittrich et al., 1998). In the basic LLBT, the linear predictor ŋ is given by


(2) $${ŋ}_{y_{jk}}=\ln\left[m\left(y_{jk}\right)\right]=\mu_{jk}+y_{jk}\left(\lambda_{j}-\lambda_{k}\right),$$

where m denotes the expected frequency of PC decisions, $\mu_{jk}$ is a nuisance parameter for the comparison jk which fixes the marginal distribution to $n_{jk}$ and $y_{jk}$ are indicator variables with value 1, if object j is preferred to k and value −1, if object k is preferred to j. The λ parameters can be transformed into worth parameters by the equation


(3) $$\pi_{j}=\exp\left(2\lambda_{j}\right)/\underset{k}{\sum }\exp\left(2\lambda_{k}\right).$$

As the ranking responses of a subject are considered simultaneously a pattern approach is used. The response pattern is defined as ***y*** = (*y*_12_, *y*_13_, …, *y*_jk_, …, *y*_*J*-1,*J*_). The expected frequency for a sequence of preferences ***y***, formulated as a loglinear model, is given as


(4) $$m\left(y\right)=m\left(y_{12},\ldots ,y_{J-1,J}\right)=np\left(y\right),$$

where *n* is the total number of respondents and p(y) denotes the probability to observe the response pattern ***y***.

To gain PC patterns of rankings, rankings are converted into a series of paired comparison decisions (Dittrich et al., 1998). Note that in the case of forced rankings (i.e., no mid-ranks), ties do not occur by definition. Rankings are transformed into a series of paired comparisons of which intransitive patterns (e.g., 1 > 2 and 2 > 3, but 3 > 1) cannot occur and as such are reduced to J! possible combinations (Dittrich et al., 2002). Model parameters are estimated using a log link and a Poisson-distributed error component. Table 1 shows the design structure of the LLBT model.

To incorporate subject covariates in BT models we used model-based recursive partitioning (MOB; Zeileis et al., 2008) to identify groups of subjects that differ in their preference rankings. The covariate space is recursively divided (partitioned) into sub-groups of subjects with varying image rankings to form a tree-structured division (Strobl et al., 2011). Each terminal node of the tree structure consists of a separate LLBT model with partition-specific model parameters. Wiedermann et al. (2021) extended the MOB BT framework to distinguish between focal independent variables (e.g., expertise status) and covariates used for recursive partitioning. The MOB LLBT model for g = 1, …, G subgroups can be written as


(5) $$\begin{array}{l} \log\left[m{\left(y_{jk}\right)}_{\left(g\right)}\right]\\ =\mu_{\left(g\right)}+\lambda_{s\left(g\right)}+y_{jk|s\left(g\right)}\left(\lambda_{j\left(g\right)}+\lambda_{js\left(g\right)}-\lambda_{k\left(g\right)}-\lambda_{ks\left(g\right)}\right), \end{array}$$

where the intercept $\mu_{\left(g\right)}$ and the main effect $\lambda_{s\left(g\right)}$ cstitute normalizing constants in subgroup g , $y_{jk|s\left(g\right)}$ gives the paired comparison decision in group *s* and partition *g* (with $y_{jk|s\left(g\right)}$ = 1 if *j* ≻ *k* and $y_{jk|s\left(g\right)}$ = −1 if *k* ≻ *j*), $\lambda_{j\left(g\right)}$ and $\lambda_{k\left(g\right)}$ denote the partition-specific object parameters for the reference group, and $\lambda_{js\left(g\right)}$ and $\lambda_{ks\left(g\right)}$ are the partition-specific effects capturing potential group differences (cf. Wiedermann et al., 2021).

Covariates are included to assess the additive impact of subjects’ characteristics on the perceived worth of image features. Students in sample I include the following covariates: the time spent on each image set (“Game Time”), gender, age, art grade, and the questions regarding artistic ability and self-perceived art skills. Sample II covariates included age, gender, time spent on each image set, and eye-tracking variables fixation time (time spent fixating image AOIs) and fixation counts (fixations lying inside image AOIs). VL expertise status (expert vs. novice) served as a focal independent variable.

Statistical analysis and model formulation were conducted with the R-package “prefmod” (Hatzinger and Dittrich, 2012), partitioning was accomplished with the R-package “partykit” (Hothorn and Zeileis, 2015). To overcome the risk of spurious tree structures a minimum node size of 40 was chosen for Sample I and a minimum of four participants for Sample II to reduce model complexity. To avoid overfitting, a post-pruning strategy based on the Akaike Information Criterion (AIC) was used to prune splits (i.e., bifurcations) that do not improve model fit (Zeileis et al., 2008). Nonparametric bootstrapping (using 1,000 resamples) was used to evaluate the stability of LLBT trees (Philipp et al., 2018). Here, we focused on selection probabilities and average cut-off (splitting) values of the pre-defined covariates. For a stable LLBT tree, selection probabilities of the initially selected covariates are expected to be close to one and average splitting values are expected to be close to the estimates obtained in the initial LLBT tree.

## Results

### Student Sample I

Table 2 shows the descriptive statistics for self-reported variables and time spent on each image set for sample I. Depending on the image set, different variables had significant impact on the preference rankings.

**Table 2:** Descriptive statistics of variables in sample I (*N* = 987 students).

Variable	Mean (SD)	
Age	15.35 (2.96)	
S1	3.63 (0.97)	
S2	3.70 (0.89)	
S3	3.33 (0.95)	
S4	3.70 (1.08)	
S5	3.26 (1.16)	
PM	2.57 (0.88)	
SO	3.20 (0.76)	
LM	2.70 (0.8)	
IM	2.05 (0.93)	
IP	2.74 (0.91)	
Art grade	1.96 (0.84)	
Mean time on…		Percentage of correct* ranking
Geometric figures	13.28 (5.45)	96%
Dogs	23.01 (10.26)	42%
Bull images	24.33 (12.71)	29%
Mondrian trees	18.16 (9.05)	36%
Salt packages	27.46 (14.49)	04%

S1-S5, self-perceived art skills; PM, photographic memory; SO, spatial orientation; LM, long-term memory; IM, imagination; IP, interest in visual puzzles. *Intended ranking: a > b > c > d > e.

Table 3 shows the worth parameters for the LLBT tree terminal node in each image set, including significant splitting covariates for sample I. Worth parameters (π) range from 0 to 1, and sum up to 1 for each node. For most image sets, exception being the “salt packages” and the “bull images,” worth parameters decline and form a slope from highest worth to lowest worth according to the intended solution for each image set.

**Table 3:** Worth parameters in each terminal node from sample I.

Sample I—students (*n* = 987)							
Image set	Term. node	Worth parameters (π) for each image					Splitting covariates
		a	b	c	d	e	
Geometric figures	*n* = 634	0.933	0.061	0.005	4.10E-04	2.00E-05	Age ≤ 15
	*n* = 259	0.921	0.069	0.007	9.00E-04	6.30E-05	Age > 15, Time ≤ 15 s
	*n* = 94	0.593	0.228	0.106	0.053	0.018	Age > 15, Time > 15 s
Dogs	*n* = 182	0.415	0.241	0.143	0.120	0.081	Time ≤ 20 s, IP ≤ 2
	*n* = 312	0.318	0.237	0.184	0.144	0.117	Time ≤ 20 s, IP > 2
	*n* = 46	0.280	0.233	0.230	0.158	0.099	Time > 20 s, IP ≤ 1
	*n* = 447	0.403	0.223	0.184	0.116	0.074	Time > 20 s, IP > 1
Bull images	*n* = 76	0.403	0.226	0.157	0.134	0.080	Time ≤ 12 s
	*n* = 911	0.585	0.186	0.091	0.099	0.038	Time > 12 s
Mondrian trees	*n* = 59	0.577	0.157	0.135	0.073	0.058	Time < 13 s, Age ≤ 14
	*n* = 117	0.509	0.176	0.182	0.081	0.053	Time < 13 s, Age > 14, LM ≤ 2
	*n* = 158	0.831	0.077	0.074	0.013	0.004	Time < 13 s, Age > 14, LM > 2
	*n* = 654	0.624	0.144	0.136	0.052	0.043	Time > 13 s
Salt-packages	*n* = 450	0.274	0.325	0.144	0.127	0.130	Male
	*n* = 495	0.325	0.347	0.113	0.109	0.107	Female

IP, interest in visual puzzles; LM, “I can remember small details in pictures” from 1 (strongly disagree) to 4 (strongly agree).

Note that at first glance, certain image sets with worth parameters close to zero would indicate no preference for any of these images. However, this is due to the continuous transformation of the BT model parameters (*λ*) into a worth parameter (π) on a scale from 0 and 1. For example, for the image set “geometric figures,” each image in the first terminal node (*n* = 634 students) is about 12–20 times more likely to be judged to be more “round” compared to the preceding image in the order “a then b then c then d then e.” Image c, ( $\hat{\pi_{c}}$ =0.005) is about 82% more likely to be chosen before image d ( $\hat{\pi_{a}}$ =0.00041) from participants in the first terminal node.

Overall, the time spent on each set and the participants’ age had the largest impact on the perceived image features. In general, faster and older student groups tend to form the steepest decline in worth parameters between each image, i.e., image preferences between each image are more clearly separated, indicating no problems in ranking the images according to the intended features. Interestingly, two self-reported visual skills “Interest in visual puzzles” (IP) and “long-term memory” (LM) were important for the judgment of abstraction (i.e., ranking images from realistic to abstract) on item set “dogs” and item set “Mondrian trees.” Here, subgroups with higher scores tended to show steeper decline in worth parameters.

**Figure 2:**
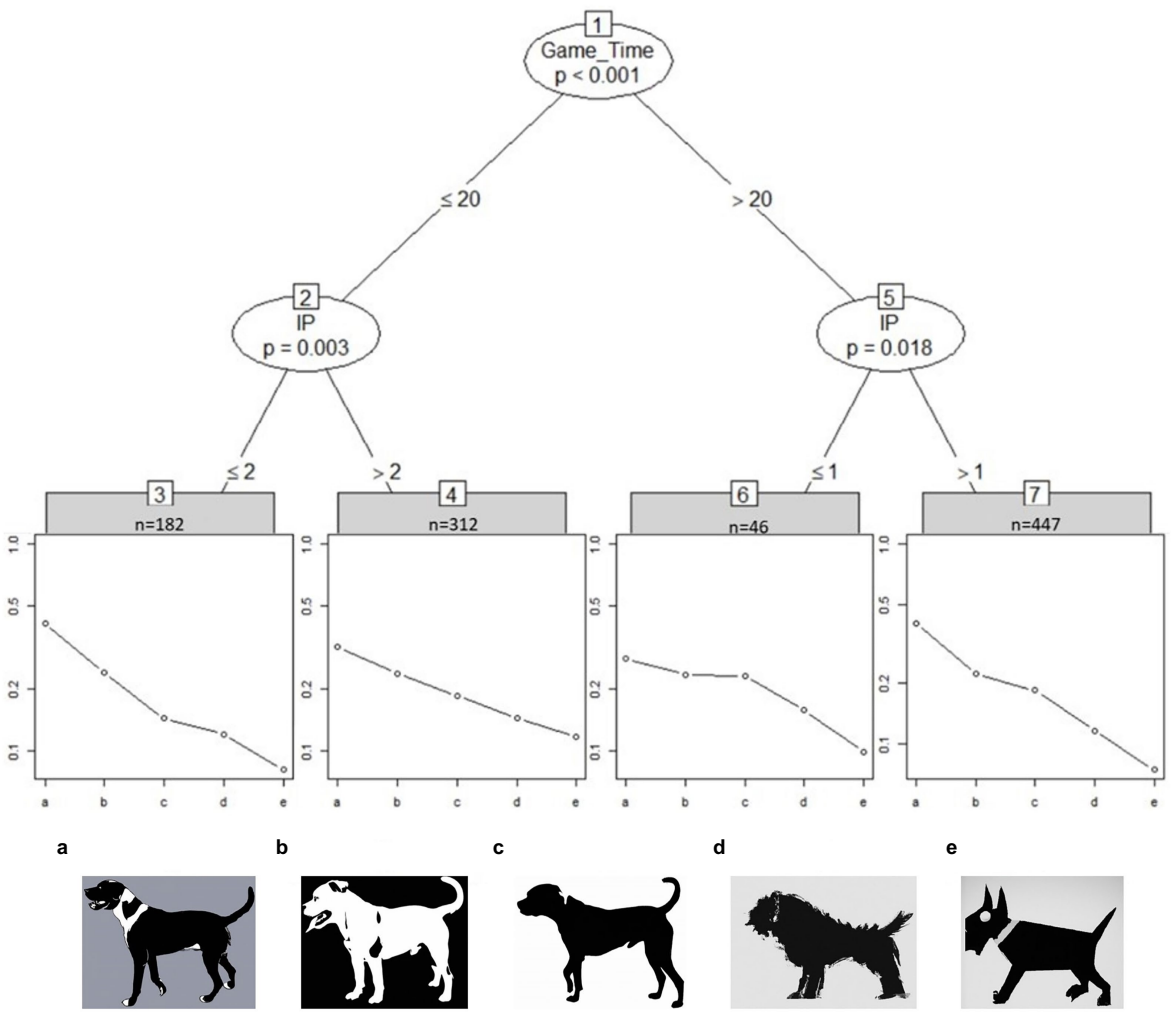
Partitioned paired comparison tree for the ranking task “dogs” in sample I. Game_Time = Time spent on image set in seconds, IP, “Interest in visual puzzles.” Fast students (<20 s) show greater differentiating skill between dog image b and c than slow students (>20 s). Self-reported IP scored greater than 1 increases the perceived differences between dog image b and c in slower student groups (node 7). Placeholder images of dogs due to copyright. Original images can be found at Billmayer (2017).

Figure 2 shows the partitioning tree for the dog images. The worth parameter is presented on a log-scale. The student sample is split between fast and slow student groups (about 50%) with one group spending less than 20 s on the image set (Game_Time < 20) and the other group going above 20 s. The gap in perceived abstraction level between dog image b and c is less noticeable for students in node 6 and 7, i.e., slower student groups show similar worth parameters between the two images. However, slower students (45%) with an interest in visual puzzles (IP > 1) perceive image c to be less realistic than image b.

**Figure 3:**
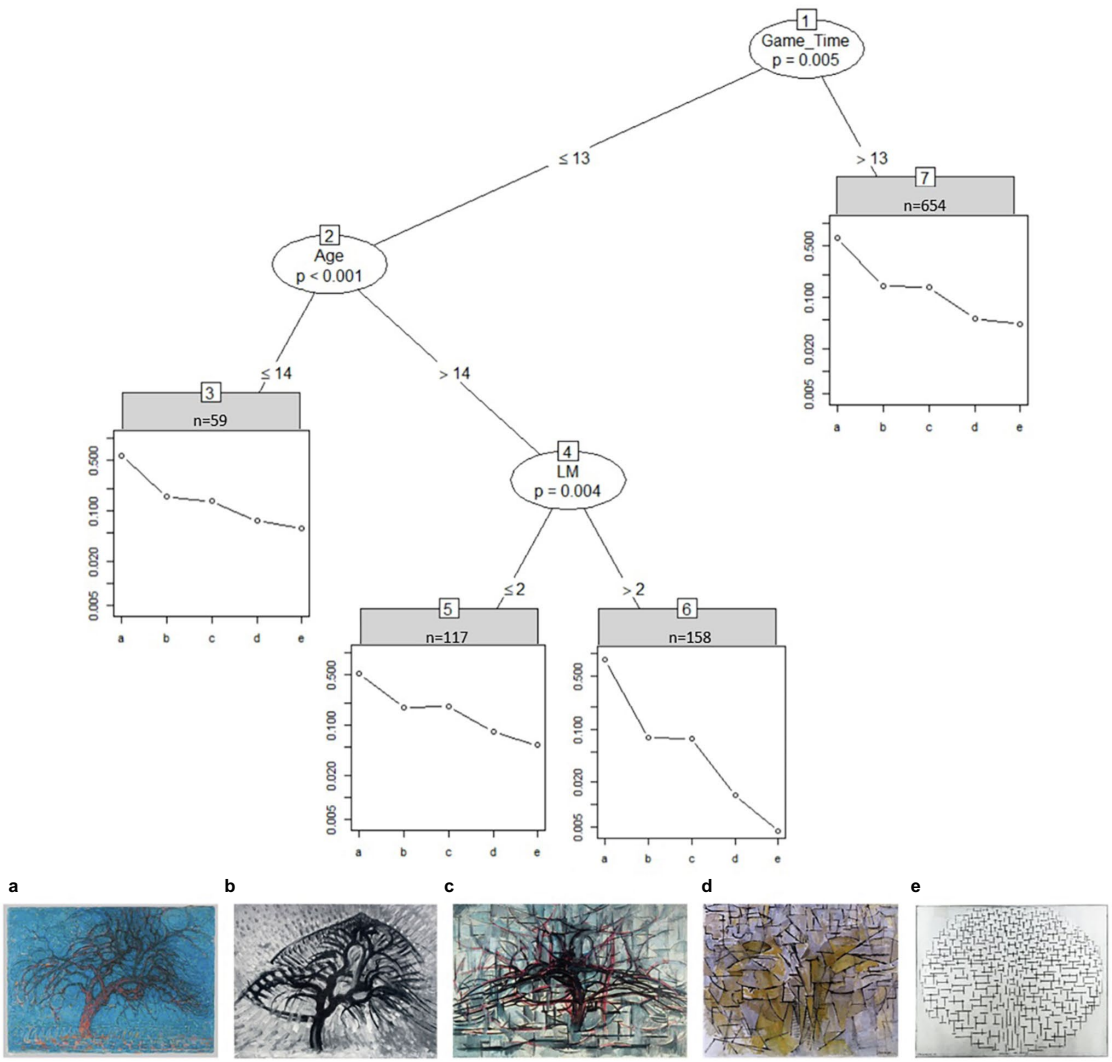
Partitioned paired comparison tree for the ranking task “mondrian trees” in sample I. Game_Time, time spent on image set in seconds; LM, “I can remember small details in pictures.” A: Piet Mondriaan, Evening, 1908–1910, oil paint on canvas, Kunstmuseum Den Haag, The Hague, inv./cat.nr17-1933/0332041 (https://rkd.nl/en/explore/images/269727); B: APiet Mondriaan, Apple tree, 1908–1909, oil paint on cardboard, Dallas Museum of Art, Dallas (Texas), inv./cat.nr 1982.26 (https://rkd.nl/en/explore/images/269740); C: Piet Mondriaan, The gray tree, 1911, oil paint on canvas, Kunstmuseum Den Haag, The Hague, inv./cat.nr156-1971/0334314 (https://rkd.nl/en/explore/images/270161); D: Piet Mondriaan, Tableau no. 4 (authentic), 1913, oil paint on canvas, Kunstmuseum Den Haag, The Hague, inv./cat.nr 159-1971 / 0334317 (https://rkd.nl/en/explore/images/270445); and E: Piet Mondriaan, Compositie 10 in zwart wit, 1915, oil paint on canvas, Kröller-Müller Museum, Otterlo (Ede), inv./cat.nr 532-15 (https://rkd.nl/en/explore/images/218082).

Figure 3 shows how time spent on the task significantly affects the way students in sample I ranked the tree images from realistic (left) to abstract (right). Most students took longer than 13 s to rank the images (*n* = 654 in node 7) and ranked images b and c close to each other. Faster students under the age of 15 also ranked the tree images according to their proposed level of abstraction (node 3). Older students with self-reported low long term visual memory skill (LM; disagreeing to the statement “I can remember small details in pictures”) rate image c to be more realistic than image b (node 5). When these students were agreeing or strongly agreeing to that statement instead (node 6) they rated the first image (a) to be nearly 11 times more realistic than the second image (b) and the last image (e) to be about three times more abstract than the fourth image (d).

**Figure 4:**
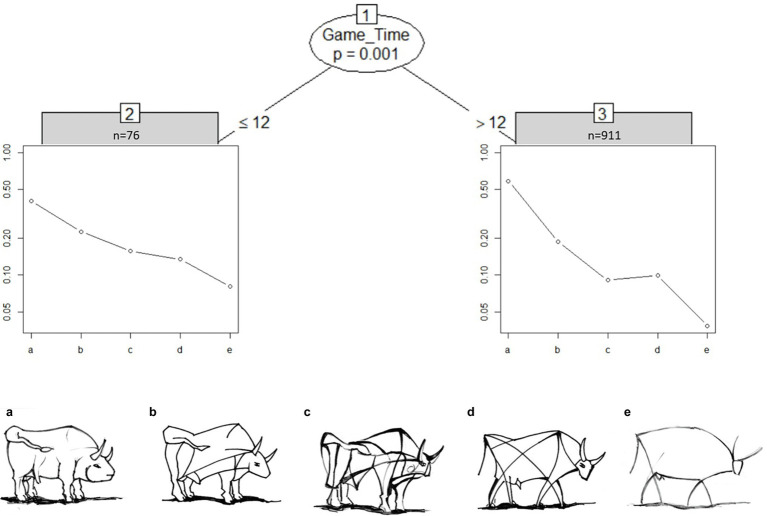
Partitioned paired comparison tree for the ranking task “bull images” in sample I. Game_Time, time spent on image set in seconds. Self-produced by author EW.

Figure 4 shows the partitioned tree for the “bull images” set for sample I. Surprisingly, most students (92%) took longer than 12 s and rated image d to be more realistic than image c. The “bull image” set is the only image set with a clear deviation from the intended solution.

**Figure 5:**
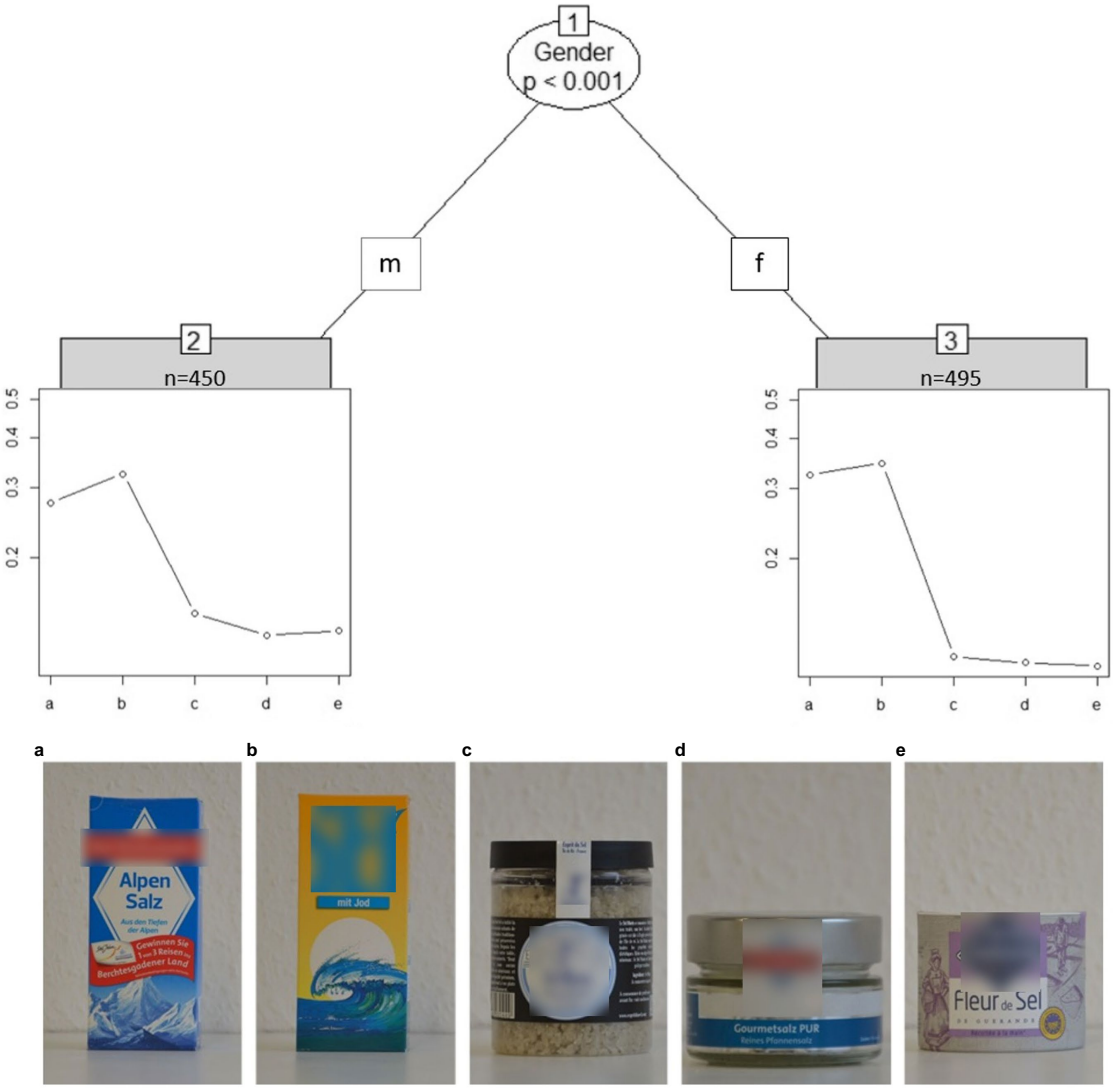
Partitioned paired comparison tree for the ranking task “salt packages” (sample I). m, male; f, female. Self-produced by author UF.

Figure 5 shows how the cost of salt packages is clearly split between images a and b vs. c, d, and e. There is also a significant difference in gender: contrary to the actual solution both genders agree b is the most expensive, but males have a smoother drop-off across a > c > d > e, whereas females rate a and b as similarly expensive, and c, d, and e as similarly cheap.

### Robustness

Stability checks were performed with a bootstrapping procedure, using 1,000 bootstrap samples. Table 4 shows the probability of splits based on each covariate in sample I and sample II. In sample I, usually, the time spent on each image set was a common splitting variable, oftentimes splitting the decision tree on each image set except for the “Geometric figures.” Students’ age had significant influence on the stimuli “bull images” and the “Mondrian trees.”

**Table 4:** Selection probabilities of splits for each variable on each image set for bootstrapping procedure on sample I and sample II.

Probability to split tree					
Variable	Geometric figures	Dogs	Bull images	Mondrian trees	Salt packages
Sample I (*n* = 987 students)					
Age	0.14	0.49	**0.60**	**0.65**	0.45
Gender	0.08	0.42	**0.79**	0.52	**0.92**
Game time	0.32	**0.98**	**0.98**	**0.92**	**0.82**
Art grade	0.16	0.33	0.35	0.39	0.31
S1	0.11	0.25	0.41	0.35	0.30
S2	0.08	0.44	0.52	0.52	0.43
S3	0.28	0.30	0.33	0.30	0.22
S4	0.12	0.27	0.45	0.55	0.42
S5	0.02	0.34	0.29	0.48	0.29
PM	0.29	0.48	0.42	0.44	0.34
SO	0.02	0.40	0.39	0.56	0.45
LM	0.27	0.34	0.42	0.44	0.33
IM	0.11	0.54	0.53	**0.61**	**0.62**
IP	0.18	**0.82**	**0.62**	**0.66**	0.45
Sample II (*n* = 49 VL-experts and novices)					
Age	0.00	0.20	0.40	0.17	–
Gender	0.00	0.01	0.00	0.00	–
Game time	0.00	0.48	0.03	0.06	–
Fix. duration a	0.00	0.19	0.07	0.05	–
Fix. duration b	0.00	0.03	0.00	0.00	–
Fix. duration c	0.00	0.04	0.00	0.05	–
Fix. duration d	0.00	0.02	0.01	0.15	–
Fix. duration e	0.00	0.06	0.01	0.00	–
Fix. count a	0.00	0.39	0.01	0.01	–
Fix. count b	0.00	0.28	0.01	0.00	–
Fix. count c	0.00	0.08	0.00	0.03	–
Fix. count d	0.00	0.09	0.05	0.01	–
Fix. count e	0.00	0.05	0.02	0.00	–

Probabilities of splits > 0.60 are marked in bold. S1–S5, self-perceived art skills; PM, photographic memory; SO, spatial orientation; LM, long-term memory; IM, imagination; IP, interest in visual puzzles; a = most realistic image to e = most abstract image.

The stability checks indicate that the results from the empirical sample I are comparable: multiple splits on the same decision tree are frequently caused by the time spent on each image set. The covariates emerging in numerous bootstrap samples exert a more stable impact on the BT model than covariates that emerge only rarely. Questionnaire items S1-S5 on self-reported artistic ability do not seem to trigger splits very often. A few exceptions are noticeable: for the “Mondrian trees” the self-reported ability to imagine (IM) was observed more often to cause a split (*M* = 0.61) in comparison to the long-term working memory (LM) variable (*M* = 0.44) that is reported in the empirical sample. IM was also nearly equally often used to split the tree of the “Salt packages” stimuli. Additionally, interest in visual puzzles (IP) was also found to split variables on the “bull images” and “Mondrian trees” (>60%), therefore might being underrepresented by the empirical sample. Bootstrapping results for the expert and novices in sample II indicate low splitting probabilities (<15%) for the eye-tracking variables. An exception being the “dogs” image set with fixations on the most realistic image splitting the tree in about 40% of the time. Lastly, the time spent on the dog images was significant in about 50% of the cases.

**Figure 6:**
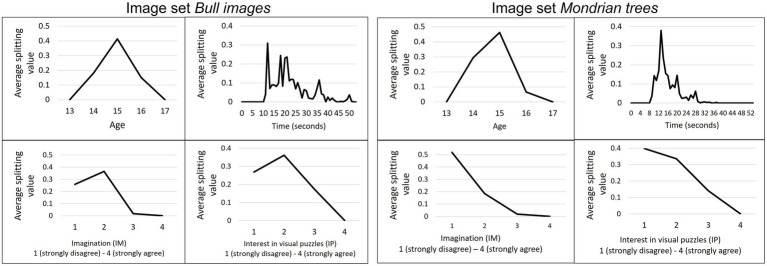
Splitting value for continuous variables in sample I. Average splitting values for the variables age, time, imagination (IM), and interest in visual puzzles (IP) on “bull images” (left) and “Mondrian trees” (right) as a result of the bootstrapping procedure in sample I.

Figure 6 shows at which values continuous variables split the tree structure as a result of the bootstrapping procedure exemplified for the “bull images” and “Mondrian trees” image set in sample I. For the variable age most splits occurred for students above or below the age of 15 years. The time spent on the task varied for the bull images with a tendency to split at 5 s or between the 10–15 s. Whereas for the “Mondrian trees” splitting peaked around the 7-s mark and then continuously dropped until reaching zero at around 22 s.

### Expert-Novice Comparison in Sample II

Worth parameters for the expert and novice comparison are listed in Table 5. Generally, experts showed a steeper, linear decline in worth parameters than novices. Subjects could not be grouped based on the number of fixations and the fixation duration on AOIs. Further, age was the only significant splitting variable on the “bull images” set.

**Table 5:** Worth parameters in each terminal node from sample II.

Sample II—VL experts and novices (*n* = 49)							
Image set	Term. node	Worth parameters (π) for each image (95% CI)					Splitting covariates
		a	b	c	d	e	
Geo-metric figures	*n* = 24 Exp	0.999 (0.99–0.99)	1.65e-09 (6.4e-10–4.2e-09)	4.65e-18 (9.5e-19–2.2e-17)	1.31e-26 (1.21e-27–1.41e-25)	2.17e-35 (9.5e-38–4.9e-33)	–
	*n* = 25 Nov	0.608 (0.48–0.85)	0.248 (0.12–0.40)	0.089 (0.04–0.19)	0.043 (0.01–0.08)	0.012 (0.004–0.06)	
Dogs	*n* = 24 Exp	0.325 (0.31–0.34)	0.255 (0.25–0.26)	0.191 (0.19–0.19)	0.135 (0.13–0.13)	0.095 (0.08–0.12)	–
	*n* = 25 Nov	0.478 (0.42–0.52)	0.197 (0.19–0.19)	0.168 (0.17–0.16)	0.100 (0.09–0.10)	0.057 (0.03–0.11)	
Bull images	*n* = 12 Exp	0.999 (0.99–0.99)	3.57e-09 (3.5e-09–3.6e-09)	1.05E-09 (1.1E-09–1.1E-09)	4.55e-10 (4.3e-10–4.7e-10)	1.62e-18 (1.2e-18–2.3e-18)	Age ≤ 28
	*n* = 22 Nov	0.999 (0.99–0.99)	3.01e-09 (2.6e-09–3.4e-09)	9.28E-10 (7.5e-10–1.1e-09)	5.25e-10 (3.9e-10–7.0e-10)	1.1e-10 (4.4e-11–2.7e-10)	
	*n* = 12 Exp	0.307 (0.29–0.32)	0.256 (0.25–0.26)	0.161 (0.16–0.16)	0.161 (0.16–0.16)	0.114 (0.09–0.15)	Age > 28
	*n* = 3 Nov	0.999 (0.99–0.99)	1.22e-08 (2.6e-07–3.4e-09)	2.54e-16 (7.5e-15–2.83-16)	5.2e-24 (7.0e-20–3.9e-28)	6.45e-32 (4.4e-30–2.7e-34)	
Mondrian trees	*n* = 24 Exp	0.748 (0.70–0.78)	0.141 (0.12–0.15)	0.085 (0.07–0.09)	0.021 (0.01–0.03)	0.006 (0.002–0.011)	–
	*n* = 25 Nov	0.999 (0.99–0.99)	2.21E-08 (1.9e-08–2.5e-08)	1.10E-08 (8.9e-09–1.4e-08)	2.58E-09 (1.4e-09–4.8e-09)	9.61E-10 (1.7e-10–5.2e-09)	

**Figure 7:**
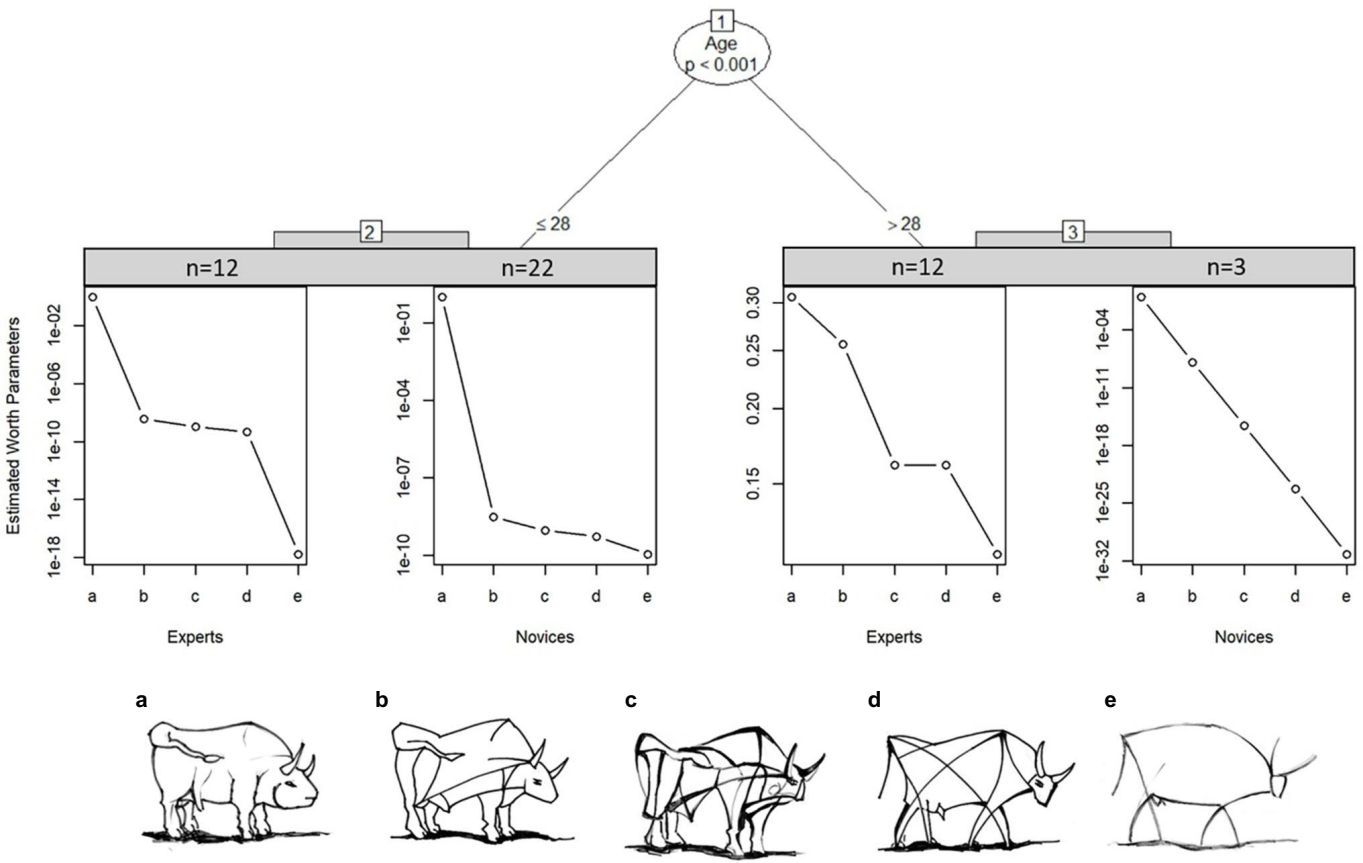
Partitioned paired comparison tree with estimated worth parameters for the ranking task “bull images” in sample II. Self-produced by author EW.

We take a closer look at how this item was perceived by the experts and novices. MOB LLBT results in in Figure 7 indicate that experts above age 28 judge bull image “c” and “d” to be very close in level of abstraction. In contrast, novices above the age of 28 estimate all bulls to have the same distance of abstraction to each other, however this may be due to the small sample size of only three novices in node 3. On the other hand, younger experts show a clear distinction between the most realistic and most abstract bull image, but differentiate only marginally between the three bull images in the middle. Novices below the age of 29 only differentiate strongly between the most realistic bull image to the rest. Generally, older participants differentiate better between the images.

Next, we focus on the distribution of attention for the preference ranking through a fixation heatmap. The mean fixation time spent on the “bull image” set in sample II was *M_Experts_* = 18.37 s (*SD* = 10.17), *M_Novices_* = 18.06 s (*SD* = 8.38). Repeated ANOVA showed that experts’ and novices’ fixation times did not significantly differ between each bull [*F*(1,47) = 0.013, n.s.]. A comparison of the distribution of fixations on each separate bull image during task completion revealed longer fixation times on bull images b, c and d compared to the most realistic (a) and most abstract (e) bull, *F*(4,188) = 28.124, *p* < 0.001.

**Figure 8:**
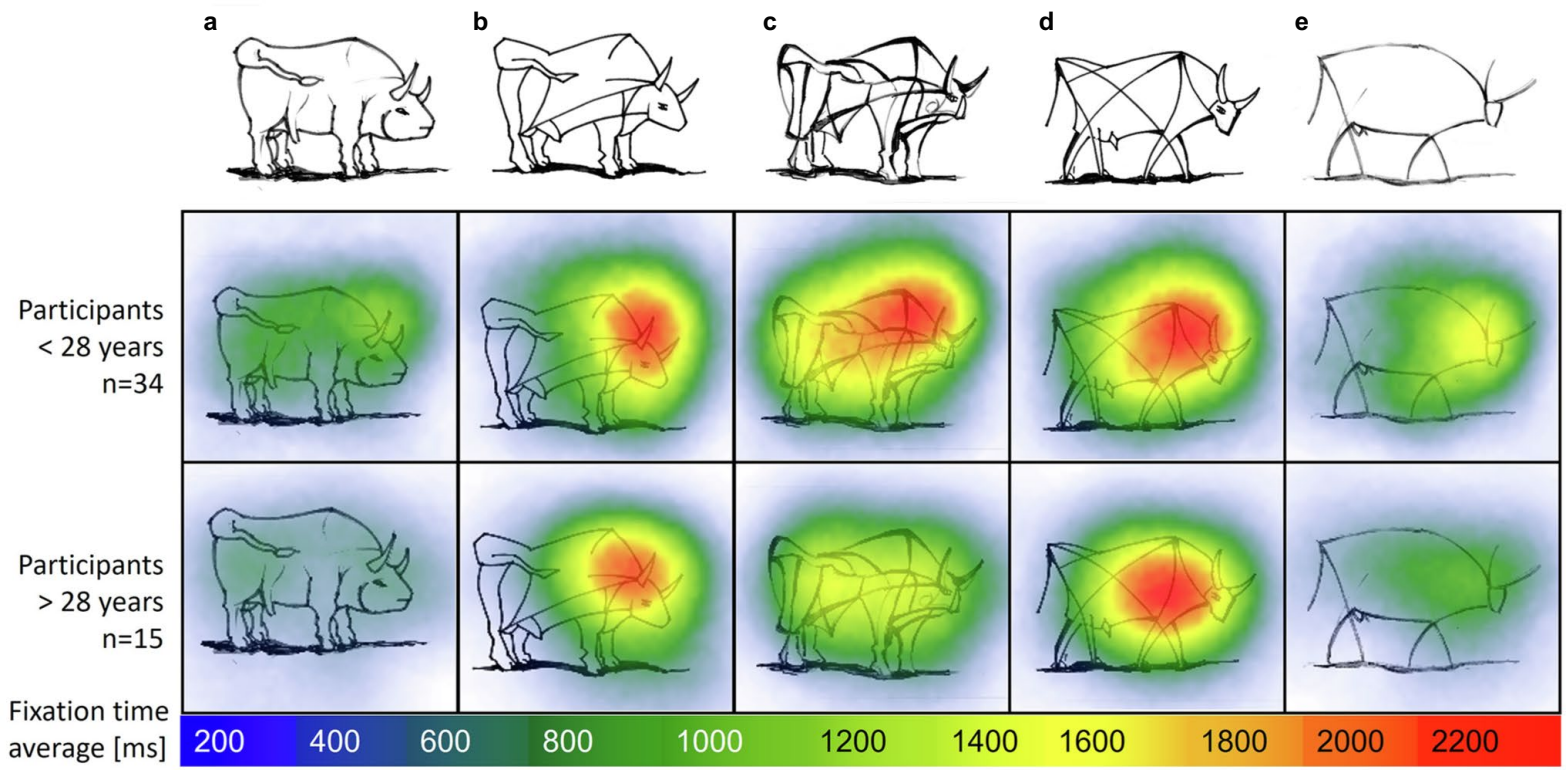
Heatmap with average fixation time on image set “bull images” by age groups. Self-produced by author EW.

Figure 8 shows a heatmap of mean fixation durations on each bull AOI from start until end of trial, supplementing the model described in Figure 7. The most abstract (right) and most realistic (left) bull image attract less attention compared to bulls of similar abstraction level. Fixation times of experts and novices was mainly spent on the bulls associated with a medium level of abstraction (b, c and d). There is a negative correlation between age and fixation time; *r*(47) = −0.36, *p* = 0.011, i.e., older participants, spend less time on images compared to younger participants. Participants below 28 years spend additional fixation time on the most abstract bull image e compared to older groups.

## Discussion

This study explored how lay students, lay adults, and visual art experts ranked more or less abstract images by applying a LLBT model to identify potential heterogeneity in visual judgments. Overall, time to complete the ranking task in combination with self-reported skills have significant influence on model parameters. In general, the longer students took to rank the images, the closer each image was ranked to the previous one, i.e., the difference in the ranked preferences between the images decreases. Students who spent more time on the task may had difficulties ranking the images the intended way. Additionally, visual skills affected the ease to differentiate between images. Interestingly, the students’ art grade did not affect the ability to rank the presented images with respect to visual abstraction. There was also no apparent classroom group effect.

The slim packaging of the “salt packages” seems to determine the perceived difference in cost. In contrast to other images, the knowledge of goods and prices is very different to the evaluation of image abstraction and is well reflected by the preference scale: the divergence between small and round vs. slim and tall salt packaging can be clearly seen in the steep drop of estimated worth parameters after image “b.” It could be hypothesized that male and female students might have different access to merchandise, which could explain the slight difference in cost perception by gender.

Furthermore, ranking abstract images such as the “bull images” revealed how similar abstraction levels of image pairs are reflected by similar worth parameters. The majority of students ranked bull image d as more realistic even though it contains less features than c. Apparently line thickness influences the perception of abstraction level for the majority of students. Also, the bull’s eye is drawn slightly more realistically in bull d in comparison to bull c, which may have influenced the ranking. Are these differences in perceived judgment of images outside the intended ranking an indication for less skilled student groups? This cannot be derived solely from the ranked preferences. Comparing this result to the sample II, revealed how VL experts above the age of 28 judged both bull images c and d to be nearly identical in abstraction level. Exploring the fixation distribution of VL experts’ and novices’ eye movements, exemplified by heatmaps, showed how images of similar abstraction level (with similar worth parameters) evoke longer fixation durations.

Students with high self-reported interest in visual puzzle solving were able to distinguish abstract images more clearly. The self-reported ability to remember small details in pictures (“working memory”) also contributed to students’ ability to rank the level of abstraction of the images, indicated by greater systematic difference (i.e., exhibiting a steeper slope across the five images) in worth parameters between each image pair. Stability checks suggest that MOB LLBT models can sufficiently detect heterogeneity of visual judgments in a large sample of students. The time students took to rank the images was a significant splitting covariate for almost all image sets. The interest in visual puzzles was the most relevant self-reported ability for ranking abstract images. Furthermore, age, for example, was a less prevalent splitting variable for the “dogs” image set but not for the “bull images” and “Mondrian trees.” This might be caused by the difference between abstraction due to signal character (dogs as information) vs. an aesthetic expression (trees and bulls as illustrations of experiences).

As seen in the results of the expert and novice comparison in sample II, VL experts were able to determine nuanced abstraction levels between images, as reflected in the similar worth parameters between image pairs. Smaller differences between certain image pairs do not necessarily reflect poorly on the ability to differentiate abstract images, but may indicate subtle image variations perceived by experts. Thus, especially when dealing with images of artwork, an interpretation by art experts and teachers is advisable.

## Limitations

A few limitations of the present study should be mentioned. Firstly, as an exploratory study by design, generalizability of empirical results is limited. Only a reduced number of item sets were presented. Causal effects of covariates over different stimuli would require an experimental design that systematically varies visual stimuli and should be tested at the end of a longer series of experimental studies. Even though the intended ranking for abstract images was moderately low (between 29% and 42%), the worth parameters did not reflect the presence of outlying responses between student groups, i.e., there was no large systematic difference in ranking order among students. Different sets of stimuli, e.g., computer generated art that controls for salience (Furnham and Rao, 2002; Shakeri et al., 2017) with a focus on a single dimensions of visual abstraction, such as composition or color (Markovic, 2010) could lead to higher variability in perceived judgment.

In comparison to other image ranking tasks (e.g., Strobl et al., 2011), an intended ordering of items was agreed upon. In the case of latent image characteristics multiple orderings may be acceptable and should be elaborated upon further (such as in the case of the “bull images”). However, a ranking assignment with heterogeneous preference patterns might indicate ambiguities with selected items. For educational assignments a clear preference ranking, with uniformly distributed worth parameters might be more desirable.

In sample II only age was found as a significant splitting variable, which might be due to low statistical power. Age of participants might also be confounded with expertise as older persons tend to have more expertise. Finally, the number of datapoints increase dramatically with the number of items for MOB LLBT models. With 5! = 120 possible PC patterns and *n* = 987 participants, the resulting input dataset consists of 118,440 observations, owing to the separate design matrices for each subject. Researchers might consider limiting the number of items during study design to reduce the design complexity.

## Conclusion

As an empirically derived observation our results suggest the following: less time spent on the visual judgments was associated with the ability to better discriminate between images of varying levels of abstraction. Abilities related to visual arts (imagination and interest in visual puzzles) seem to support this discriminative ability demonstrated by our participants.

In contrast to measurements of visual judgment with visual analog scales (e.g., the AAA instrument by Chatterjee et al. (2010)), ranking tasks lets participants compare multiple images at once. BT trees then can be used in various educational settings, e.g., art assignments where exact iconicity between two images is unknown. Judging images of more varying complexity (see García et al., 1994 for an early attempt to measure icon complexity) could be a next step in the construction of future test batteries on VL.

The presented modelling approach allows one to quantify the distance between images on a standardized latent scale. Here, BT models do not rely on the assumption of equidistant response categories. The latent metric scale is derived from ordinal (ranking) data to capture the perceived between-group differences of visual judgment. The perceived distance between each image (e.g., level of abstraction) can then be used to identify closely related and, therefore, hard-to-differentiate objects. Such objects could subsequently be discussed and analyzed in art class.

## Author’s Note

The results of this study were used by author MT to fulfil some of the requirements for the doctoral degree program at the University of Regensburg.

## Data Availability Statement

The raw data supporting the conclusions of this article will be made available by the authors, without undue reservation.

## Ethics Statement

The studies involving human participants were reviewed and approved by the Ethics Committee of Research of the Leibniz Institute for Research and Information in Education, Frankfurt am Main (DIPF, 01JK1606A). Written informed consent to participate in this study was provided by the participants’ legal guardian/next of kin.

## Author Contributions

MT, UF, and KR designed the study. MT and EW selected and prepared the stimuli. MT conducted the field work and eye-tracking experiments, performed the statistical analysis with input on data interpretation by WW, UF, and MG, and prepared the manuscript. All authors reviewed the article and approved the submitted version.

## Funding

This work was supported by the German Federal Ministry of Education and Research (BMBF) under grant number 01JK1606A.

## Conflict of Interest

The authors declare that the research was conducted in the absence of any commercial or financial relationships that could be construed as a potential conflict of interest.

## Publisher’s Note

All claims expressed in this article are solely those of the authors and do not necessarily represent those of their affiliated organizations, or those of the publisher, the editors and the reviewers. Any product that may be evaluated in this article, or claim that may be made by its manufacturer, is not guaranteed or endorsed by the publisher.
